# Effects of prophylactic constant-rate infusion of norepinephrine or phenylephrine on neonatal outcomes during caesarean section

**DOI:** 10.1186/s40001-025-03761-3

**Published:** 2026-01-04

**Authors:** Wenhui Tao, Jinfeng Bao, Qing Wang, Yufang Xie, Shun Li, Zicheng Wang, Shoufeng Zhu, Linghui Huang, Yan Yang, Xun Xu, Pengcheng Zhu, Panpan Ding, Jieli Tian, Xiaofen Liu, Haifeng Cao, Wei Liu, Shuwen He, Wensheng He, Ye Zhang, Xianwen Hu

**Affiliations:** 1 Department of Anesthesiology and Perioperative Medicine, The Second Affiliated Hospital of Anhui Medical University, Key Laboratory of Anesthesiology and Perioperative Medicine of Anhui Higher Education Institutes, Anhui Medical University, No.678 Furong Road, Economic and Technological Development Zone, Hefei City, 230601 Anhui Province China; 2 Department of Anesthesiology, Hefei Hospital Affiliated to Anhui Medical University (The Second People’s Hospital of Hefei), No. 246 Heping Road, Hefei, 230011 Anhui Province China

**Keywords:** Norepinephrine, Phenylephrine, Cesarean section, Spinal anesthesia, Neonatal outcomes

## Abstract

**Background:**

Prophylaxis of norepinephrine (NE) at a constant rate has been demonstrated to be as efficacious as prophylaxis of phenylephrine (PE) at equivalent doses for the prevention of maternal hypotension during cesarean section. Nevertheless, the impact of prophylactic infusion of NE or PE at a constant rate on pregnant women on fetal outcomes remains to be elucidated.

**Methods:**

90 women scheduled for caesarean section under spinal or combined spinal–epidural anesthesia were randomly assigned to either the NE or PE group. The “study drug” (NE or PE) was administered intravenously at a rate of 15 mL/h from the time of injection of subarachnoid solution until the time of delivery of the fetus. Fetal umbilical artery (UA) blood was collected for blood gas analysis. The primary outcome of the study was base excess, and the incidence of fetal acidosis (Defined as base excess < 6 mmol/l) and blood glucose levels were also assessed.

**Results:**

The UA base excess mean (standard deviation) was not different from the NE group, − 1.6 (2.6) versus − 2.4 (2.9) in the PE group (*P* = 0.223). The incidence of fetal acidosis was 4.7% (NE) versus 14.3% (PE), with no statistically significant difference (*P* = 0.308). However, fetal blood glucose levels were significantly lower in the NE group, 3.16 (0.43) versus 3.43 (0.60) in the PE group (*P* = 0.019).

**Conclusions:**

Prophylactic infusion of equivalent doses of NE at a constant rate resulted in fetal base excess values and an incidence of acidosis comparable to that of PE. However, a lower fetal UA blood glucose value was observed in the NE group, a finding that warrants further investigation.

## Key points

- Question: Do prophylactic infusions of phenylephrine and norepinephrine differentially affect umbilical artery blood BE and blood glucose after fetus delivery?
- Finds: Prophylactic infusion of equivalent doses of phenylephrine and phenylephrine does not affect the incidence of base excess and incidence of acidosis, while there are differences in UA blood glucose.
- Meaning: Phenylephrine may be more beneficial in cases of fetal growth restriction, maternal concomitant diabetes mellitus, and other conditions that may lead to an increased incidence of fetal hypoglycemia.

## Introduction

Norepinephrine (NE) and phenylephrine (PE) both have alpha-adrenergic receptor agonist effects and are commonly used to maintain blood pressure during spinal anesthesia for caesarean section [1, 2]. NE exhibits weak beta-adrenergic receptor agonist activity, which counteracts the baroreflex-induced decreases in heart rate and cardiac output that occur during unopposed stimulation of vascular alpha-adrenergic receptors. NE has been used as a continuous infusion and intermittent boluses and is as effective as PE for the management of spinal anesthesia-induced hypotension [3–6].

Several studies have demonstrated that the administration of NE or PE to maintain blood pressure in pregnant women during cesarean section does not result in a differential effect on fetal outcomes [7, 8]. However, the lack of uniformity in dosing regimens and standards, particularly for single injections, is significantly influenced by the anaesthetist's clinical experience. This may introduce bias in results and hinder the dissemination of the regimens employed. In recent years, the adoption of prophylaxis of NE or PE at a constant rate has seen a marked increase in popularity, primarily due to the simplification of the infusion regimen, the elimination of the need for complex calculations and rate adjustments, and the high regard in which it is held by anaesthetists [9, 10].

The objective of the present randomized controlled, double-blind trial was to compare the effects of constant-rate NE infusion and constant-rate PE infusion on the neonatal outcomes in women undergoing caesarean section under spinal or combined spinal–epidural anesthesia. The prophylactic infusion of NE or PE at a constant rate was designed to improve the applicability and reproducibility of the study results to normal clinical practice. We hypothesized that there would be no significant difference in fetal acid–base balance, as measured by umbilical artery base excess, between women receiving a prophylactic constant-rate infusion of norepinephrine and those receiving a prophylactic infusion of phenylephrine. The primary outcome of this study was fetal umbilical artery (UA) blood base excess. Secondary outcomes included the incidence of fetal acidosis and UA blood glucose levels.

## Methods

This trial was conducted at the Second People’s Hospital of Hefei from July 2025 to September 2025 in the form of a randomized, double-blind trial involving women undergoing caesarean section under spinal anesthesia or combined spinal–epidural anesthesia. The research protocol was endorsed by the Institutional Ethics Committee of the Second People’s Hospital of Hefei and registered on ClinicalTrials.gov with the identifier No. ChiCTR2500105396. It adhered to the principles outlined in the Declaration of Helsinki and strictly followed the Consolidated Reporting Standards for Clinical Trials throughout the implementation phase [11].

At the preoperative consultation, patients were screened and evaluated. All participants provided their written informed consent before enrollment. This clinical trial enrolled 90 singleton and full-term pregnant women who were classified as American Society of Anesthesiologists (ASA) physical status class II, had uncomplicated pregnancies, and were normotensive. The exclusion criteria were as follows: participants under 18 years of age, individuals with known allergies to PE or NE, and those with fetuses suspected of having malformations. Participants were given the right to withdraw from the study at any stage without any penalty. During the perioperative period, the anesthesiologist and the obstetrician worked in close collaboration to evaluate and address any unfavorable occurrences. Their joint evaluation also determined whether the patient should continue to participate in the study. The subjects were randomly assigned to either the PE group or the NE group in a 1:1 ratio using a computer-generated random number sequence by an independent statistician who was not involved in the study enrollment or intervention. The randomization sequence was created using random block sizes of 4 and 6 to ensure balanced group allocation while concealing the allocation sequence from the investigators. The group allocation information was sealed in opaque envelopes.

Before the initiation of the surgical procedure, the dilution process was carried out by a nurse. A series of identical 50 ml syringes containing NE at a concentration of 8 μg/ml and PE at a concentration of 100 μg/ml were diluted using a 5% dextrose solution. Each of these syringes was clearly labelled with the identifier “study solution” along with the corresponding randomization code. Intravenous access was established for every patient through an 18G intravenous catheter, and they were then administered intravenous fluids. Specifically, before the surgery began, a rapid intravenous infusion of 5 mL/kg of Ringer’s lactate solution was given over a period of 15 min. Following this initial infusion, the rate of the intravenous drip was adjusted to 6 mL/kg per hour and maintained at this rate until the completion of the procedure. The ambient temperature within the operating room was set to range between 22 and 24 °C. Upon entering the operating theatre, the patient’s vital signs were meticulously monitored. Throughout the surgical procedure, the hemodynamic parameters were manually recorded at 5-min intervals.

Spinal or combined spinal–epidural (CSE) anesthesia was performed with the patient in the lateral decubitus position at the L_2_–L_3_ or L_3_–L_4_ interspace. For spinal anesthesia, 1.5 ml of 1.0% ropivacaine hydrochloride was injected intrathecally via a 25-gauge needle. For the CSE technique, the epidural space was first identified using an 18-gauge Tuohy needle. A long 25-gauge spinal needle was then passed through the Tuohy needle to enter the subarachnoid space. After confirmation of free flow of cerebrospinal fluid, the same intrathecal dose of 1.5 ml of 1.0% ropivacaine was administered. An epidural catheter was threaded 3–5 cm into the epidural space. The catheter was secured but not used for intraoperative medication in any patient. The surgery was only allowed to start when the block levels reached the T_4_ dermatomal level.

The “constant-rate” infusion refers to the prophylactic baseline rate, which was subject to pre-defined adjustments based on hemodynamic parameters for patient safety. The criteria for hypotension and hypertension were systolic blood pressure (SAP) < 80% and > 120% of baseline, respectively. In this research, specific guidelines were established for the infusion of the “study drug” according to fluctuations in SAP and heart rate. When the SAP rose to more than 120% of the baseline value, the infusion rate of the “study drug” was adjusted down to 7.5 mL/h. If the SAP escalated further and exceeded 130% of the baseline, the infusion of the drug was immediately stopped. On the contrary, when the SAP decreased to less than 80% of the baseline, the infusion rate was increased to 30 mL/h. The aim was to maintain the SAP within the range of 80–120% of the baseline. Regarding the heart rate, if the heart rate dropped below 60 beats per minute (bpm) and the SAP stayed between 80 and 120% of the baseline, the drug infusion was terminated. In situations where the heart rate was lower than 55 bpm and the SAP was less than 80% of the baseline, or when the absolute heart rate fell below 50 bpm, 0.5 mg of atropine should be administered intravenously.

A pediatrician, who was unaware of the specific subgroup assignments, collected heparinized samples from the triple-clamped of the UA. These samples were subsequently analyzed for base excess (BE), pH, HCO_3_, PCO_2_, PO_2_, blood glucose (GEM Premier 3500, Instrumentation Laboratory, USA). The incidence of fetal acidosis defined as umbilical artery base excess greater than 6 mmol/l was also assessed in two groups. The threshold of BE <  − 6 mmol/L was selected to define fetal acidosis, aligning with definitions commonly used in comparative studies of vasopressors during cesarean delivery to identify meaningful differences in fetal metabolic status. Raw data were recorded directly on the case report form. The primary outcome was BE in group NE versus group PE.

### Data collection

The preoperative data set incorporated the maternal age, weight, height, and gestational duration, along with the baseline values of systolic blood pressure (SAP), diastolic blood pressure (DBP), and heart rate (HR). Once the fetus was delivered, UA blood was collected and subjected to blood gas analysis. The postoperative data set encompassed details, such as the total volume of fluids administered, the amount of hemorrhage, the urine output, the length of time the surgery lasted, and the incidence of hypotension or reactive hypertension.

### Statistical analysis

This study was designed as a randomized comparative trial. The sample size was calculated to detect a prespecified difference in the primary outcome (umbilical artery base excess) between the two groups, not to test for non-inferiority or equivalence. Our previous study (unpublished) using PE and NE showed that UA base excess (mean ± standard deviation) in fetuses was − 2.7 ± 2.3 (PE) and − 1.1 ± 2.0 (NE), respectively.

In a study conducted by Apoorva Singh et al., the median umbilical artery base excess was found to be − 5.4 and − 6.95 in the NE and PE groups, respectively [7]. To detect a difference of 1.6 in the median umbilicalartery base excess, the sample size was estimated in this trial using PASS software (version 15, USA). With *α* = 0.05, *β* = 0.10, and a sample size of 45 per group based on a 10% dropout rate, this trial was planned to enroll 90 eligible patients.

The data were analyzed using the statistical software SPSS (version 16.0, IBM Corp, USA). The data analyst remained blinded to the group assignments (which were coded as Group A and Group B) until all analyses were completed. A *p* value of less than 0.05 was considered statistically significant. Continuous data were assessed for normality using the Kolmogorov–Smirnov test and expressed as mean ± standard deviation (SD) or median (inter-quartile range), as appropriate. Normally distributed data were analyzed using Student's *t* test, whereas non-normally distributed data were analyzed using the Mann–Whitney *U* test. Categorical data were compared using a *χ*^2^ test or Fisher’s exact test, as appropriate, and expressed as numbers and percentages. Analyses of all secondary outcomes were considered exploratory in nature, and as such, no adjustment for multiple comparisons was performed. A post hoc power analysis was conducted for the incidence of fetal acidosis using the observed difference between groups.

## Results

**Fig. 1:**
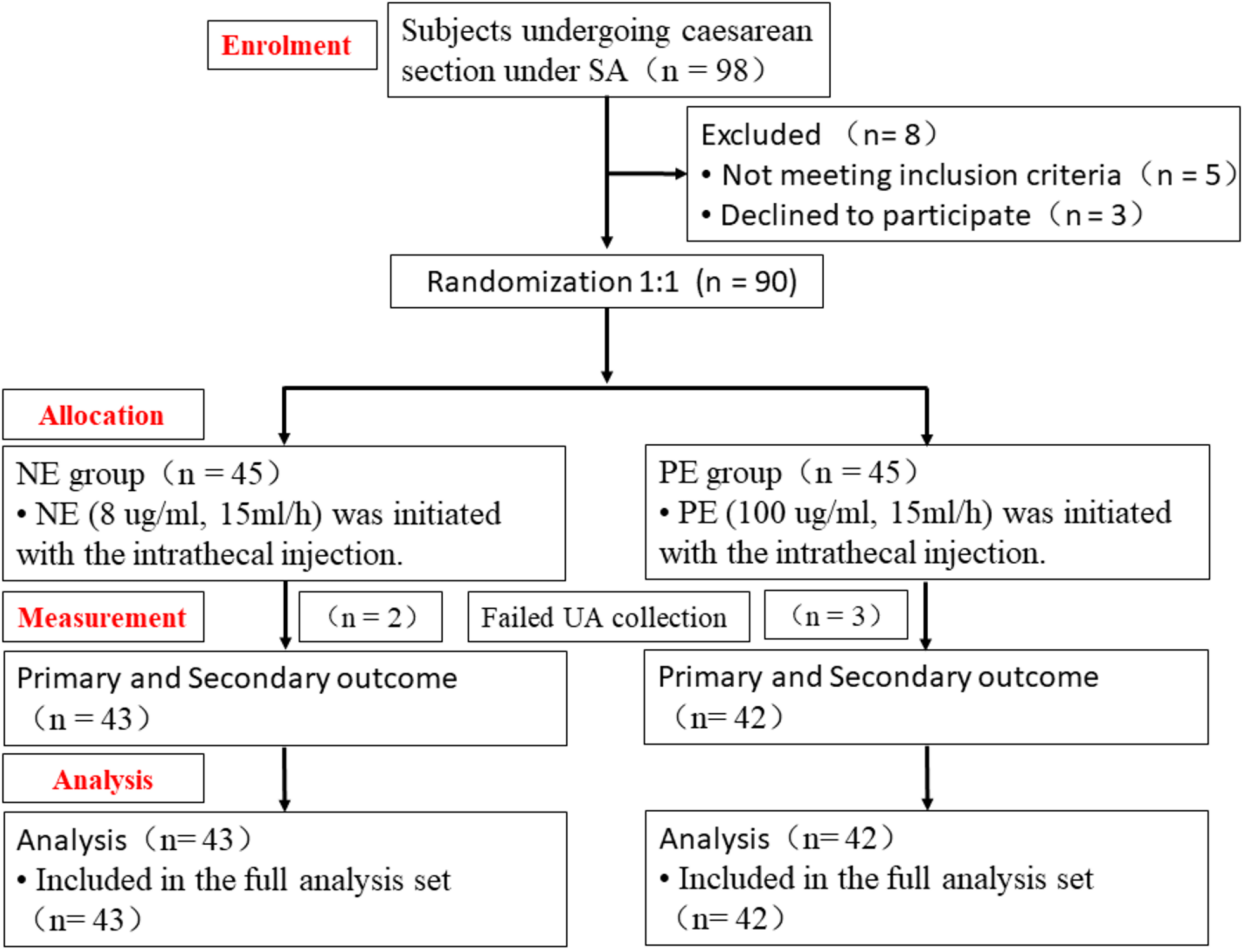
CONSORT flow diagram. SA, spinal anesthesia. UA, umbilical artery

In the groups administered PE and NE, three and two cord blood samples failed, respectively. Therefore, the number of cases that participated in blood gas analysis was 42 and 43 in the PE and NE groups, respectively. The flow chart of the study, which details the process of parturient enrolment, allocation and analysis, is presented in Fig. 1. Baseline characteristics and intraoperative data are shown in Table 1. The characteristics of the two groups were well-balanced. A significantly lower risk was observed in the NE group for both bradycardia (9.3% vs. 28.6%; *P* = 0.042) and the requirement for atropine rescue (4.7% vs. 21.4%; *P* = 0.048) compared to the PE group. No differences were observed between the two groups regarding demographic parameters, baseline hemodynamics, surgical times, the occurrence of hypotension, or reactive hypertension. The total vasopressor dose infused was significantly higher in group PE compared with group NE.

**Table 1:** Parturient demographic characteristics and intraoperative data

Parameters	PE (*n* = 42)	NE (*n* = 43)	*P* value
Age (yr)	32.3 [21–39]	30.9 [24–39]	0.123
Weight (kg)	72.1 ± 9.3	73.1 ± 9.0	0.600
Height (cm)	159.3 ± 4.9	159.7 ± 4.3	0.649
Gestational (weeks)	38.8 ± 0.8	38.7 ± 1.1	0.908
Baseline SAP (mmHg)	117.0 ± 12.7	119.7 ± 11.5	0.304
Baseline DAP (mmHg)	76.4 ± 5.2	77.0 ± 5.8	0.581
Baseline HR (bpm)	85.7 ± 6.8	84.7 ± 7.3	0.527
Fluid administered (mL)	953.6 ± 108.4	960.5 ± 104.4	0.766
Duration of surgery (min)	56.7 ± 12.0	54.4 ± 10.8	0.354
Blood loss (mL)	300(300–300)	300(300–300)	0.178
Urine volume (mL)	200(200–200)	200(100–200)	0.076
Incidence of hypotension, *n*/*N* (%)	8/42 (19.0%)	9/43 (20.9%)	0.828
Reactive hypertension, *n*/*N* (%)	3/42 (7.1%)	3/43 (7.0%)	0.976
Incidence of bradycardia, *n*/*N* (%)	12/42 (28.6%)	4/43 (9.3%)	0.042*
Atropine administration, *n*/*N* (%)	9/42 (21.4%)	2/43 (4.7%)	0.048*
Total vasopressor dose (induction-to-delivery, μg)	750.6 ± 64.1	66.7 ± 7.531	< 0.001*

Values are mean ± standard deviation, mean [range], median (inter-quartile range). *P < 0.05 was considered a significant difference between groups

**Fig. 2:**
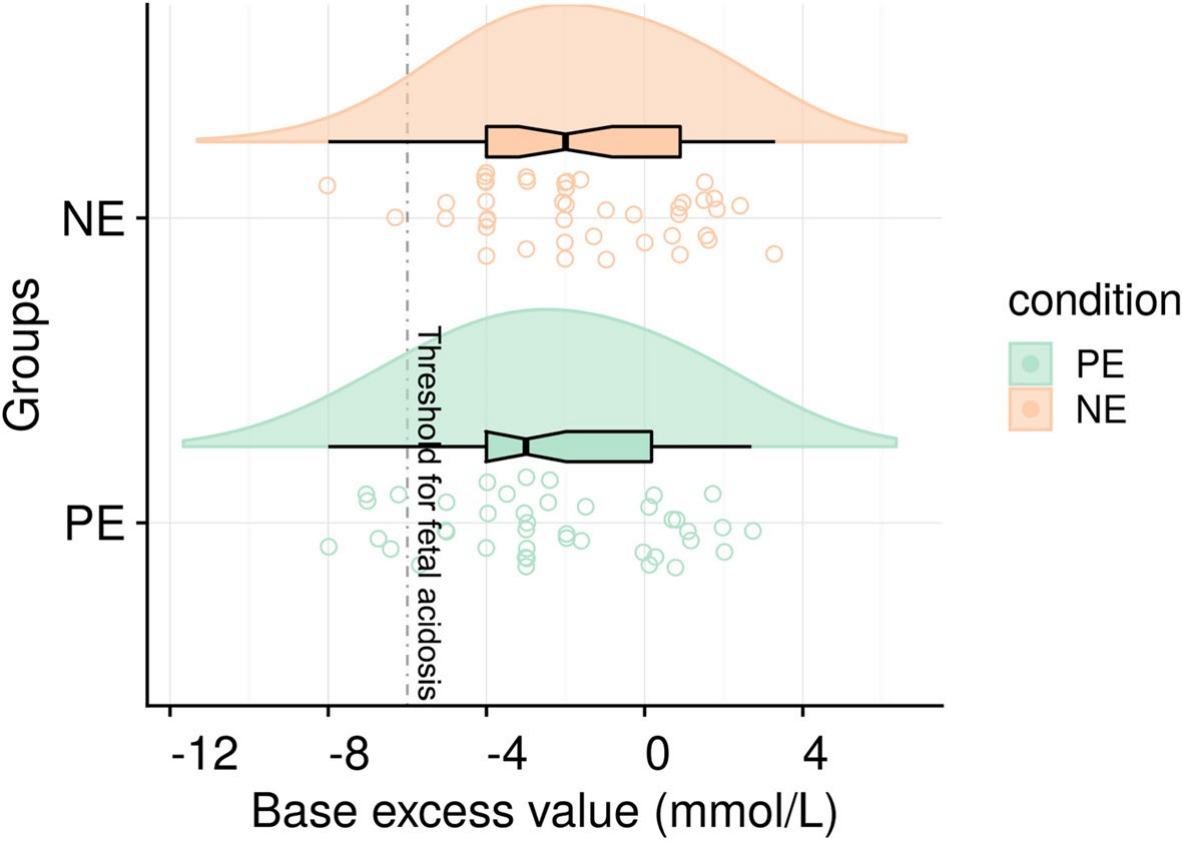
Fetal UA base excess in the two groups. Box plots show the median and interquartile ranges at 25% and 75%. Raincloud plots show the distribution of BE; the dashed line corresponds to a value of − 6 mmol/L(threshold for fetal acidosis) (NE: *n* = 43, PE: *n* = 42)

**Fig. 3:**
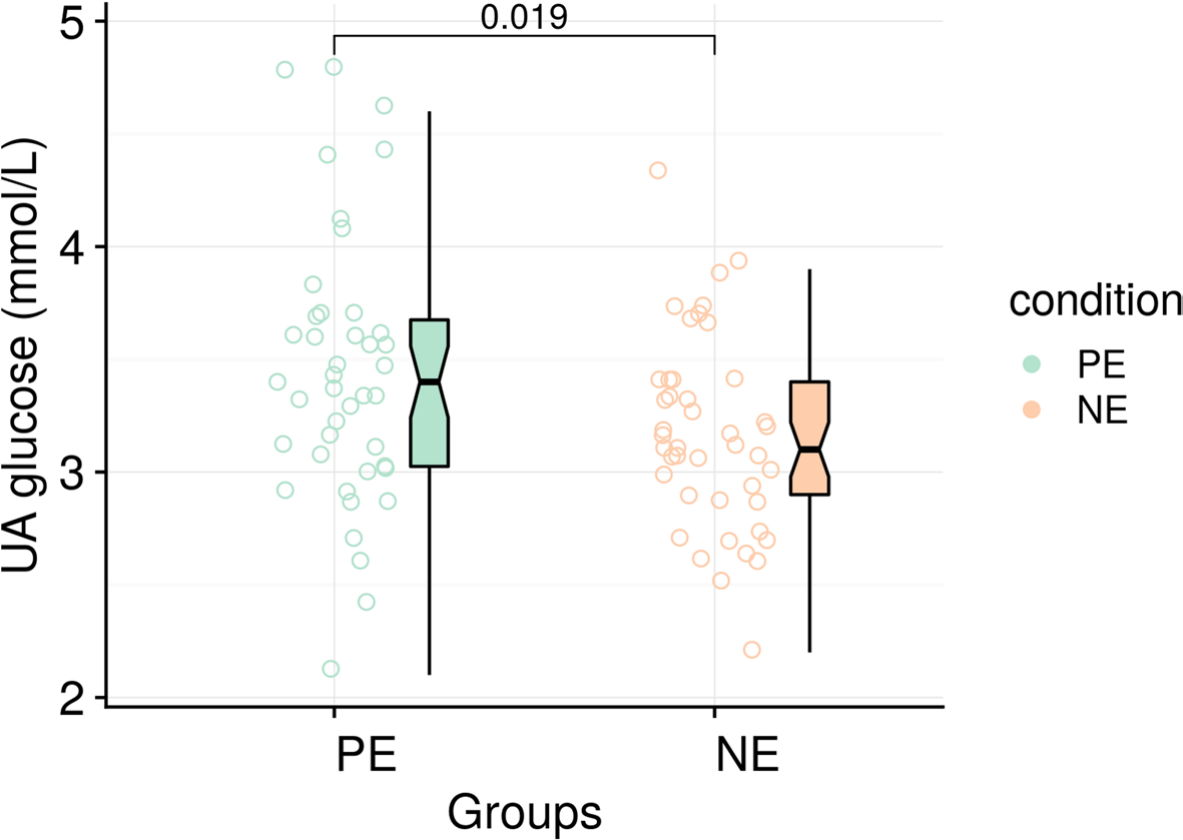
Fetal UA blood glucose in the two groups. Box plots, median and interquartile range ranges of 25% and 75%. Plots show the distribution of blood glucose (NE: *n* = 43, PE: *n* = 42)

The mean blood BE was not different from group NE: − 1.6 (95% CI − 2.44 to − 0.84) compared with group PE: − 2.4 (95% CI − 3.27 to − 1.48). The absolute mean difference was 0.8 mmol/L (95% CI − 0.45 to 1.91;* P* = 0.223, Fig. 2). The UA pH, PO_2_, PCO_2_, HCO_3_^−^ and incidence of fetal acidosis were comparable between two groups (Table 2). It should be noted that this significant finding for blood glucose in group NE: 3.16 (95% CI 3.03–3.29) compared with group PE: 3.43 (95% CI 3.25–3.62; *P* = 0.019, Fig. 3), a secondary outcome, is exploratory in nature, as the study was not powered for this endpoint and no adjustment for multiple comparisons was performed. This finding should, therefore, be interpreted as hypothesis-generating for future research.

**Table 2:** Comparison of neonatal UA blood gas analysis results between the two groups

Parameters	PE (*n* = 42)	NE (*n* = 43)	*P* value
PH	7.284 ± 0.040	7.289 ± 0.025	0.463
PO_2_ (mmHg)	14.0 (11.0–18.3)	14.0 (12.0–17.0)	0.815
PCO_2_ (mmHg)	53.8 ± 5.5	53.6 ± 5.4	0.915
HCO_3_^−^ (mmol/L)	24.3 ± 2.7	24.8 ± 2.5	0.366
Base excess (mmol/L)	− 2.4 ± 2.9	− 1.6 ± 2.6	0.223
Incidence of fetal acidosis	6 (14.3%)	2 (4.7%)	0.264
Blood glucose (mmol/L)	3.43 ± 0.60	3.16 ± 0.43	0.019*

Values are mean ± standard deviation, median (inter-quartile range) or number (%). *P < 0.05 was considered a significant difference between groups

## Discussion

The present randomized controlled trial aimed to compare the effects of constant-rate infusions of NE and PE on fetal outcomes during cesarean delivery under spinal or combined spinal–epidural anesthesia. For the primary outcome, the absolute mean difference in UA base excess between the NE and PE groups was 0.8 mmol/L (95% CI − 0.45 to 1.91). Although this difference was not statistically significant, its magnitude is small and the confidence interval indicates that the true difference is unlikely to be clinically important for neonatal well-being, as it falls well within the range of normal physiological variation in healthy term neonates. The results showed that neither NE nor PE significantly disrupted the fetal acid–base balance. However, a notable distinction was observed in fetal UA blood glucose levels, with lower values recorded in the NE group.

UA base excess, the primary outcome of this study, indicates the effects of fetal hypoxemia, anaerobic metabolism, and non-volatile acid accumulation, which is the metabolic component of academia [12]. pH, a commonly used indicator of acid–base balance, reflects both the metabolic and respiratory components and the body's physiological compensatory mechanisms, which can generally be maintained within a certain range. UA pH did not differ between the two groups when anesthetists infused or pushed 6 μg/ml NE or 100 μg/ml PE to prevent or treat hypotension based on their clinical experience [8], which is consistent with the results of the present study and confirms that a constant-rate prophylactic of NE or PE is equally safe for the fetus.

In the treatment of maternal hypotension during caesarean section, the intravenous administration of 100 μg of PE or 8 μg of NE had no effect on neonatal prognosis [7]. A fixed-rate infusion of either 4 μg/min NE or 50 μg/min PE effectively treated hypotension. However, NE demonstrated a more favorable neonatal acid–base balance [9], which is inconsistent with the present study. This may be due to a higher infusion rate accentuating the agonistic effect of NE on the β-receptor, which better maintains placental blood flow. The prophylactic infusion of 5 μg/min of NE resulted in higher base excess values than the infusion of 100 μg/min of PE [13], which is also inconsistent with the present study. This may be since the equivalent dose of NE to PE used in this study was the recognized 13:1 [2, 14] rather than the 20:1 suggested by some studies [15]. These results imply that, whether continuous infusion or single bolus, larger doses or higher rates of NE, as opposed to PE, appear to be more effective in exerting the beta-agonist effect of NE, which is also more beneficial in maintaining fetal acid–base homeostasis. While our adaptive protocol resulted in variable total drug exposure, it reflects a standardized clinical strategy for vasopressor support. This enhances the practical applicability of our findings, though it should be considered when making direct numerical comparisons with studies using different dosing regimens.

The present study employed a deliberately lower dose prophylactic strategy for both vasopressors compared to some prior studies [15]. We acknowledge this less common dosing approach, which was chosen to effectively prevent hypotension while minimizing the risk of side effects associated with higher doses, such as pronounced bradycardia from phenylephrine. Our findings demonstrate that at these lower prophylactic doses, the acid–base benefit of NE over PE is not evident, suggesting that such an advantage may be dose-dependent. The use of a standardized rescue protocol ensured maternal and fetal safety, supporting the viability of this lower dose strategy as a clinical option that balances efficacy with a potentially improved hemodynamic profile. While our adaptive protocol resulted in variable total drug exposure, it reflects a standardized clinical strategy for vasopressor support. This enhances the practical applicability of our findings, though it should be considered when making direct numerical comparisons with studies using different dosing regimens.

In the present study, lower UA blood glucose concentrations were found in the NE group. Potential explanations might include an independent negative correlation between NE concentrations and systemic insulin clearance [16]. Furthermore, it is plausible that sustained high concentrations of NE in vivo result in a prolonged glucose-stimulated hyperinsulinotropic response [17, 18]. However, it is crucial to emphasize that these are hypotheses, and this study was not designed to confirm them. The clinical manifestations of hyperinsulinemic hypoglycemia can be explained by an imbalance between maternal insulin secretion and insulin-stimulated glucose utilization due to elevated catecholamine levels [19]. The difference could also be influenced by PE’s pure α1-agonist profile potentially reducing maternal insulin secretion more than NE, leading to relative maternal hyperglycemia and subsequent transplacental transfer affecting fetal levels [20, 21]. This speculative mechanism requires validation in future studies. The higher, though not statistically significant, incidence of fetal acidosis in the PE group might be related to its potential vasoconstrictive effects on the uteroplacental circulation, but this also remains conjectural. The intriguing finding regarding fetal glucose levels naturally leads to consideration of its implications for women with gestational diabetes mellitus (GDM). However, it is imperative to state clearly that our trial excluded patients with GDM or pre-existing diabetes. Therefore, any potential clinical implications for this specific population are purely hypothetical and must be interpreted with extreme caution, necessitating dedicated future investigation.

The strengths of this study include the randomized controlled design, which minimizes bias, and the use of UA blood gas analysis, which provides a direct assessment of fetal acid–base status. However, there are several limitations to consider.

First, it was designed and analyzed as a comparative trial, not as a formal non-inferiority or equivalence study, as no such margin was pre-defined. Therefore, the finding of no significant difference in the primary outcome should not be interpreted as evidence of equivalence between the two vasopressor regimens. The sample size, although adequate for detecting differences in base excess, may have been underpowered to detect smaller differences in secondary outcomes, such as fetal acidosis. A post hoc power analysis revealed that the study was underpowered (power = 28%) to detect the observed difference in the incidence of fetal acidosis, underscoring that this negative finding should be interpreted with caution. Second, this study lacks detailed maternal hemodynamic trend data (e.g., mean arterial pressure and heart rate over time), which could provide further insight into the interplay between maternal physiology and fetal outcomes. Third, the short study period and relatively small sample size limit the generalizability of our findings and the ability to detect rare adverse events. Fourth, the collection of umbilical venous blood samples in addition to arterial samples could have enriched the interpretation of fetal metabolite status. Fifth, the constant-rate infusion protocol, while standardized, may not reflect the dynamic dosing adjustments often required in clinical practice. Future studies could explore the effects of variable-rate infusions or bolus administration of these vasopressors on fetal outcomes. Finally, the lack of a control group that did not receive prophylactic vasopressor infusion means that the observed lower umbilical artery blood glucose in the norepinephrine group cannot be definitively attributed to a glucose-lowering effect of norepinephrine, a glucose-elevating effect of phenylephrine, or a combination of both effects. This exploratory finding should be interpreted as generating a hypothesis for future investigation. The long-term implications of the observed differences in fetal glucose levels and acidosis incidence remain unclear and longer term follow-up are warranted to assess the potential extended clinical implications of the observed differences in fetal glucose metabolism. Although the study was double-blinded and managed via a strict hemodynamic protocol, we cannot entirely rule out the possibility that clinicians might have inferred the vasopressor assignment based on differential heart rate effects. However, we believe this risk was minimized by the protocolized management, and crucially, the primary outcome was assessed by a fully blinded investigator. It is crucial to distinguish our short-term, prophylactic vasopressor use from the prolonged, high-dose infusions in critical care, which are linked to endothelial dysfunction and hypoperfusion [22]. The safety concerns from sustained exposure in critically ill patients are, therefore, not applicable to our healthy obstetric population.

In conclusion, our findings suggest that constant rate infusions of NE and PE are equally effective in maintaining fetal acid–base balance during cesarean delivery under spinal or combined spinal–epidural anesthesia. An exploratory finding of this study was lower umbilical artery blood glucose levels in the norepinephrine group. This observation, while requiring validation in studies designed specifically for this endpoint, and interpreted in the context of our exploratory analysis without multiple comparison adjustment, could suggest a differential impact of vasopressors on fetal glucose metabolism.

## Data Availability

The original datasets obtained in the study are available from the corresponding author.

## Abbreviations

ASA: American Society of Anesthesiologists
CS: Cesarean section
HR: Heart rate
PE: Phenylephrine
NE: Norepinephrine
SA: Spinal anesthesia
SAP: Systolic blood pressure
BE: Base excess
UA: Umbilical artery
